# PPAR-α Agonist Suppresses Expression of Immune Mediators in B Cells in a Murine Model of Systemic Lupus Erythematosus

**DOI:** 10.3390/ph19040642

**Published:** 2026-04-18

**Authors:** Haneen A. Al-Mazroua, Hussain N. Alhamami, Mushtaq A. Ansari, Ahmed Nadeem, Sabry M. Attia, Saleh A. Bakheet, Abdulaziz M. S. Alsaad, Hatun A. Alomar, Alaa A. Alanteet, Sheikh F. Ahmad

**Affiliations:** Department of Pharmacology and Toxicology, College of Pharmacy, King Saud University, Riyadh 11451, Saudi Arabia

**Keywords:** systemic lupus erythematosus, WY14643, B lymphocytes, inflammatory mediators, PPAR-α agonist

## Abstract

**Background/Objectives:** Systemic lupus erythematosus (SLE) is a chronic autoimmune disorder characterized by immune dysregulation that leads to widespread inflammation and damage across multiple organs. B lymphocytes play a vital role in SLE, with abnormal development and activation leading to autoreactive antibody production and immune complex formation, which damages tissues. **Methods:** The PPARα agonist WY14643 has anti-inflammatory effects in various inflammatory conditions, including CNS diseases. We investigated whether WY14643 decreases inflammatory mediator production in CD45R^+^ cells in the MRL/lpr mouse model of SLE. Flow cytometry was used to evaluate WY14643’s impact on the expression of IFN-γ, IL-6, iNOS, MCP-1, IL-1α, IL-2, Notch-1, Notch-3, GITR, and NF-κB p65 in splenic CD45R^+^ B cells. Additionally, we assessed the effect of WY14643 on the mRNA levels of these markers in the kidney using RT-PCR. **Results:** WY14643 decreased inflammatory markers such as CD45R^+^IFN-γ^+^, CD45R^+^IL-6^+^, CD45R^+^iNOS^+^, CD45R^+^MCP-1^+^, CD45R^+^IL-1α^+^, CD45R^+^IL-2^+^, CD45R^+^Notch1^+^, CD45R^+^Notch3^+^, CD45R^+^GITR^+^, and CD45R^+^NF-κB p65^+^ in splenic cells from MRL/lpr mice. Furthermore, WY14643 also lowered mRNA expression of IFN-γ, IL-6, iNOS, MCP-1, IL-2, IL-1α, Notch-1, Notch-3, GITR, and NF-κB p65 in the kidney. **Conclusions:** This study shows that WY14643 inhibits the production of inflammatory mediators and significantly reduces autoimmune features, including kidney inflammation, in MRL/lpr mice. Our results indicate that WY14643, a PPAR-α agonist, could be a potential therapy for lupus nephritis.

## 1. Introduction

Systemic lupus erythematosus (SLE) is a chronic autoimmune disease characterized by involvement of multiple systems and organs, recurrent episodes of remission and relapse, and numerous autoantibodies [1,2]. The etiology of SLE is complex and involves multiple factors, including heredity, sex hormones, and environmental factors (such as viral and bacterial infections) [3]. It is well established that autoantibody production, immune complex formation, and inflammatory responses driven by multiple cytokines contribute to the development of SLE [4]. B cells play a key role in the pathogenesis of autoimmune diseases [5]. Previous evidence indicates that B lymphocyte hyperactivity is a pathogenic event in SLE and contributes to the development of kidney disease [6]. B cells exhibit disrupted signaling pathways, leading to abnormal activation and differentiation in SLE [7]. Furthermore, there are imbalances in regulatory B cells, with a tendency toward pro-inflammatory B cells [8]. Additionally, B cells contribute to the progression of SLE by presenting antigens to self-reactive T cells and secreting inflammatory cytokines, which promote inflammation in multiple organs, including the kidneys, lungs, heart, and skin [9].

IFN-γ plays an important role in the pathogenesis of lupus nephritis [10]. In renal tissues, IFN-γ expression was significantly higher in patients with lupus nephritis and correlated with the activity of pathological lesions. IFN-γ promotes B cell class switching and stimulates the production of pathogenic autoantibodies in SLE [11]. Patients with SLE have higher levels of IFN-γ than controls [12]. IFN-γ has been implicated in the pathogenesis of SLE [13,14]. Among the immunological mediators implicated in SLE, IL-6 has attracted significant attention due to its diverse biological roles. In patients with SLE, IL-6 levels correlate with disease activity, renal involvement, and abnormal B and T cell function [15]. At the molecular level, IL-6 promotes B cell differentiation and autoantibody production, as well as Th17-cell expansion, a crucial immunopathological process in SLE [16,17]. Patients with SLE have higher serum IL-6 levels, and IL-6 is also found in cerebrospinal fluid [18].

Studies have shown that MCP-1 plays a significant role in fibrosis across multiple organs [19]. A recent study reported a substantial increase in MCP-1 in patients with lupus nephritis (LN) [20]. Alzawawy et al. previously reported that increased MCP-1 levels correlate with LN disease severity [21]. iNOS is closely linked to the initiation of inflammation [22]. Moreover, evidence indicates that iNOS is an inflammatory inducer that promotes LN progression [23,24]. Increased iNOS expression is linked to kidney damage in patients with SLE [25] and in mouse models [26].

Notch signaling is a highly conserved pathway across species that facilitates intercellular interactions and regulates the differentiation, growth, and development of various cell types [27,28]. Essential for tissue and organ development during embryogenesis, Notch signaling continues to influence developmental processes after birth and is associated with human diseases [29]. Research suggests that blocking Notch1 signaling could be an effective therapy for SLE [30]. One study also observed that patients with active SLE exhibit notably lower levels of Notch-1 mRNA and protein than healthy controls [31]. A recent study showed that abnormal Notch signaling has been implicated in several autoimmune conditions [32,33]. Another study demonstrated that disturbed Notch signaling plays a key role in the development of SLE [34]. A recent investigation further confirmed that inhibiting Notch1 signaling significantly reduces pristane-induced lupus in mice [35].

Research shows that the nuclear receptor PPAR-α influences inflammation across various tissues, including the vascular wall, heart, nervous tissue, lung, gut, and liver [36,37]. In one study, WY14643 significantly reduced the secretion of pro-inflammatory cytokines [38]. Activation of PPAR-α by WY14643 helps alleviate systemic LPS-induced acute lung injury [39] and protects cortical neurons from damage caused by pro-inflammatory mediators [40]. Additionally, WY14643 has been shown to lower inflammatory markers in experimental periodontitis [41]. It can also suppress pro-inflammatory responses in microglia [42]. Moreover, WY14643 has been found to reduce reactive oxygen species production triggered by perfluorododecanoic acid in rat liver [43]. WY14643 improves renal preservation in kidneys subjected to chronic perfusion [44].

The MRL/lpr strain is among the most well-established spontaneous models of SLE and is frequently used in lupus-related neuropsychiatric research. MRL/lpr mice naturally develop an autosomal recessive lymphoproliferative (lpr) mutation affecting the Fas gene [45]. A deletion in the Fas gene’s intron causes abnormal splicing of Fas mRNA [45] and results in the absence of Fas protein expression [46]. The disease in MRL/lpr mice closely resembles human SLE. As in humans, where a significant gender disparity exists (9:1 female-to-male ratio), female MRL/lpr mice tend to develop a more severe form of the disease. Hence, in the present study, we investigated the effects of WY14643 on immune response mediators in B cells and explored the potential mechanisms underlying its therapeutic effects in MRL/lpr mice. Clarifying the therapeutic potential of this innovative approach will advance the development of effective treatments for SLE-related kidney damage, ultimately improving patient care and outcomes.

## 2. Results

### 2.1. The PPAR-α Agonist WY14643 Decreases IFN-γ and IL-6 Expression

**Figure 1:**
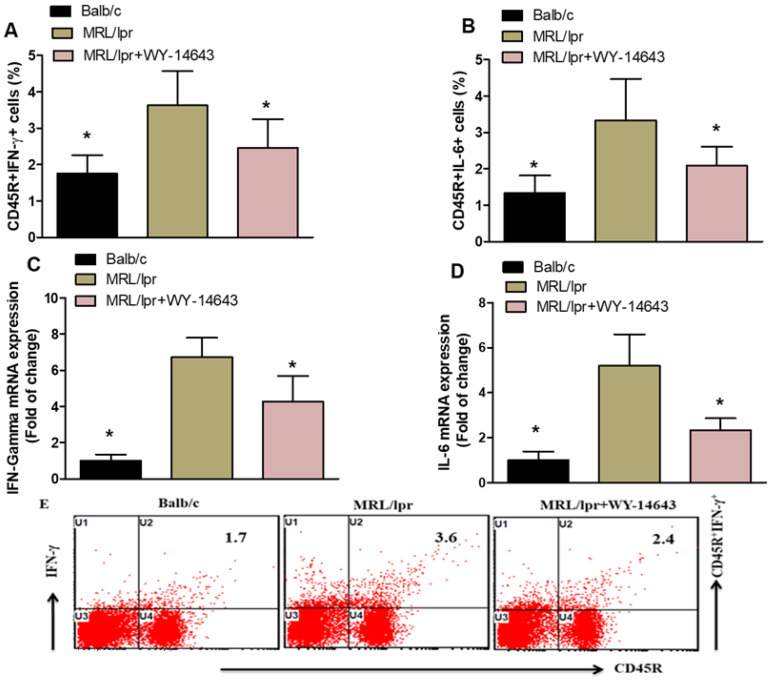
(**A**,**B**). Effects of WY14643 on IFN-γ- and IL-6-expressing CD45R^+^ cells were analyzed by flow cytometry in splenocytes. (**C**,**D**) mRNA levels of IFN-γ and IL-6 were analyzed by RT-PCR in kidney tissue from MRL/lpr mice treated with WY14643. Cells were gated on forward- and side-scatter dot plots to determine the percentage of IFN-γ- and IL-6-expressing CD45R^+^ B cells in the spleen. (**E**) Representative Dot plots of a mouse from each group. MRL/lpr mice were treated with 10 mg/kg WY14643 intraperitoneally daily for eight weeks, whereas Balb/c mice (wild-type control) received a vehicle. The graphs represent the mean score ± SD of six mice per group. Statistical significance was determined as * *p* < 0.05.

Flow cytometry analysis revealed that WY14643 decreases the expression of inflammatory markers in MRL/lpr mice. The number of CD45R^+^ cells producing IFN-γ and IL-6 was significantly lower in the spleens of WY14643-treated mice than in vehicle-treated controls (Figure 1A,B). Additionally, RT-PCR of kidney tissue showed reduced levels of IFN-γ and IL-6 mRNA in WY14643-treated mice compared with vehicle-treated mice (Figure 1C,D). These results suggest that WY14643 suppresses inflammatory mediators in MRL/lpr mice, indicating that activating PPAR-α may help reduce inflammation and cytokine production in this SLE model.

### 2.2. PPAR-α Agonist WY14643 Treatment Decreases iNOS and MCP-1 Expression

**Figure 2:**
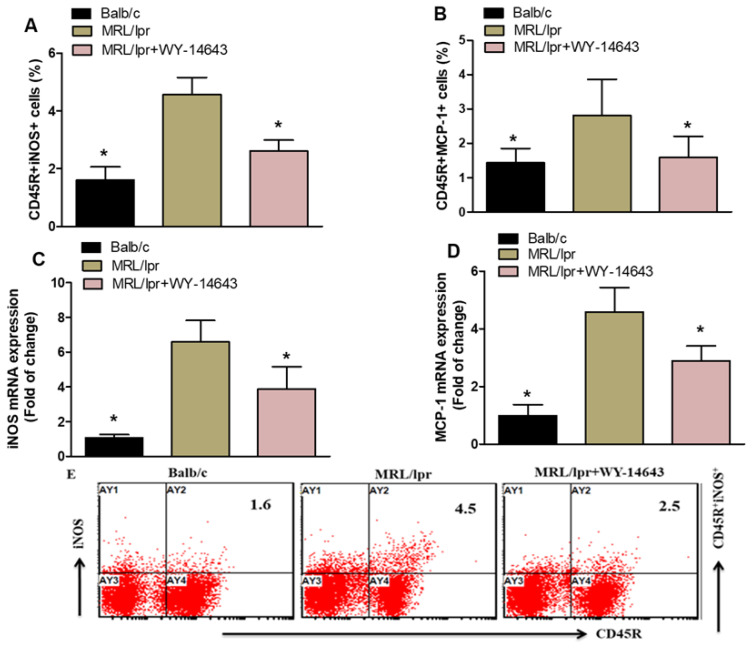
(**A**,**B**). Effects of WY14643 on iNOS- and MCP-1-expressing CD45R^+^ cells were analyzed by flow cytometry in splenocytes. (**C**,**D**) mRNA levels of iNOS and MCP-1 were analyzed by RT-PCR in kidney tissue from MRL/lpr mice treated with WY14643. Cells were gated on forward- and side-scatter dot plots to determine the percentage of iNOS- and MCP-1-expressing CD45R^+^ B cells in the spleen. (**E**) Representative Dot plots of a mouse from each group. MRL/lpr mice were treated with 10 mg/kg WY14643 intraperitoneally daily for eight weeks, whereas Balb/c mice (wild-type control) received a vehicle. The graphs represent the mean score ± SD of six mice per group. Statistical significance was determined as * *p* < 0.05.

We investigated the effects of WY14643 on CD45R^+^ cells expressing iNOS and MCP-1 in MRL/lpr mice. The counts of CD45R^+^iNOS^+^ and CD45R^+^MCP-1^+^ cells were markedly lower in WY14643-treated MRL/lpr mice than in vehicle-treated mice (Figure 2A,B). RT-PCR results also showed reduced levels of iNOS and MCP-1 mRNA in the kidneys of WY14643-treated MRL/lpr mice compared with vehicle-treated mice (Figure 2C,D). These findings demonstrate that WY14643 treatment decreases iNOS and MCP-1 expression in MRL/lpr mice.

### 2.3. Effects of WY14643 on IL-1α- and IL-2-Expressing CD45R^+^ Cells

**Figure 3:**
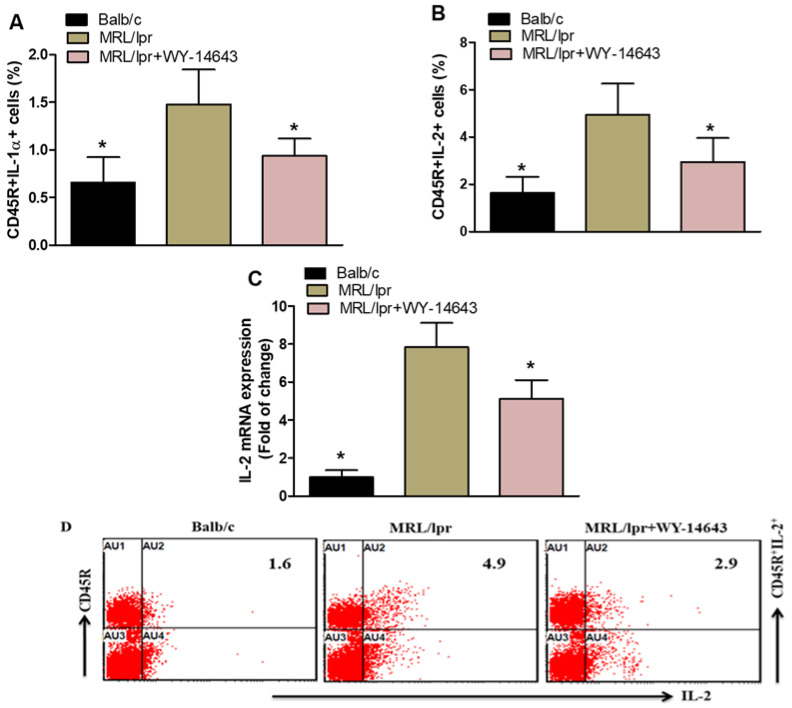
(**A**,**B**). Effects of WY14643 on IL-1α- and IL-2-expressing CD45R^+^ cells were analyzed by flow cytometry in splenocytes. (**C**) mRNA levels of IL-1α and IL-2 were analyzed by RT-PCR in kidney tissue from MRL/lpr mice treated with WY14643. Cells were gated on forward- and side-scatter dot plots to determine the percentage of IL-1α- and IL-2-expressing CD45R^+^ B cells in the spleen. (**D**) Representative Dot plots of a mouse from each group. MRL/lpr mice were treated with 10 mg/kg WY14643 intraperitoneally daily for eight weeks, whereas Balb/c mice (wild-type control) received a vehicle. The graphs represent the mean score ± SD of six mice per group. Statistical significance was determined as * *p* < 0.05.

Flow cytometry revealed a significant decrease in CD45R^+^IL-1α^+^ and CD45R^+^IL-2^+^ cells in WY14643-treated mice compared with vehicle-treated controls (Figure 3A,B). Additionally, IL-2 mRNA levels in kidney tissue were markedly lower in WY14643-treated mice than in vehicle-treated controls (Figure 3C). These results suggest that WY14643 inhibits inflammatory mediator production in the SLE mouse model.

### 2.4. The PPAR-α Agonist WY14643 Inhibits the Notch-1 and Notch-3 Signaling

**Figure 4:**
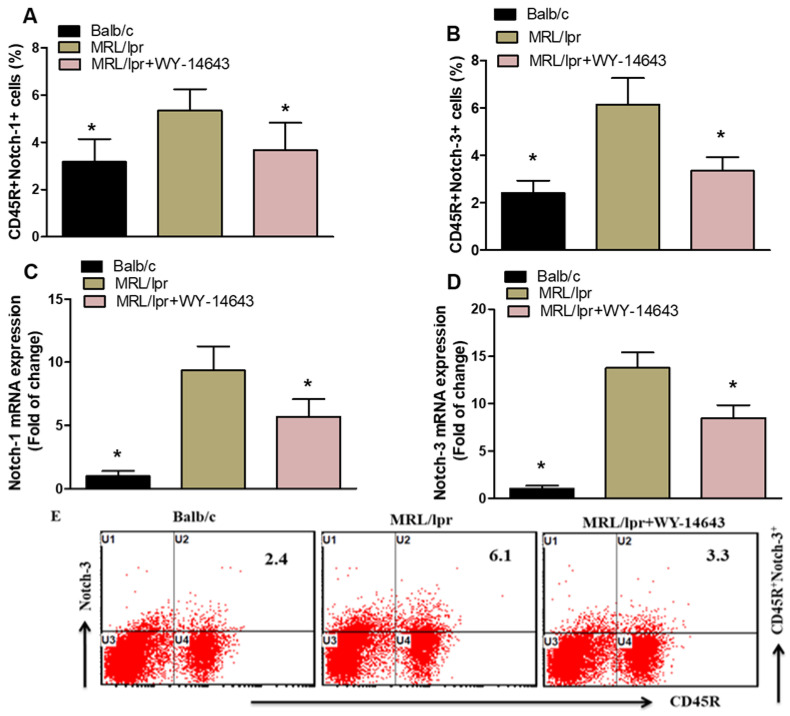
(**A**,**B**). Effects of WY14643 on Notch-1- and Notch-3-expressing CD45R^+^ cells were analyzed by flow cytometry in splenocytes. (**C**,**D**) mRNA levels of Notch-1 and Notch-3 were analyzed by RT-PCR in kidney tissue from MRL/lpr mice treated with WY14643. Cells were gated on forward- and side-scatter dot plots to determine the percentage of Notch-1- and Notch-3-expressing CD45R^+^ B cells in the spleen. (**E**) Representative Dot plots of a mouse from each group. Treated MRL/lpr mice received 10 mg/kg WY14643 intraperitoneally daily for eight weeks, whereas Balb/c mice (wild-type control) received a vehicle. The graphs represent the mean score ± SD of six mice per group. Statistical significance was determined as * *p* < 0.05.

The study further analyzed Notch1 and Notch3 expression in splenic CD45R^+^ B cells. Treatment with WY14643 significantly reduced Notch1 and Notch3 levels in CD45R^+^ cells from MRL/lpr mice compared with vehicle-treated controls (Figure 4A,B). Consistent with the protein results, WY14643 also markedly decreased Notch1 and Notch3 mRNA levels in kidney tissue from MRL/lpr mice (Figure 4C,D). These results indicate that WY14643 effectively inhibits Notch1 and Notch3 signaling pathways.

### 2.5. WY14643 Decreases the Expression of GITR and NF-κB p65 in CD45R^+^ Cells

**Figure 5:**
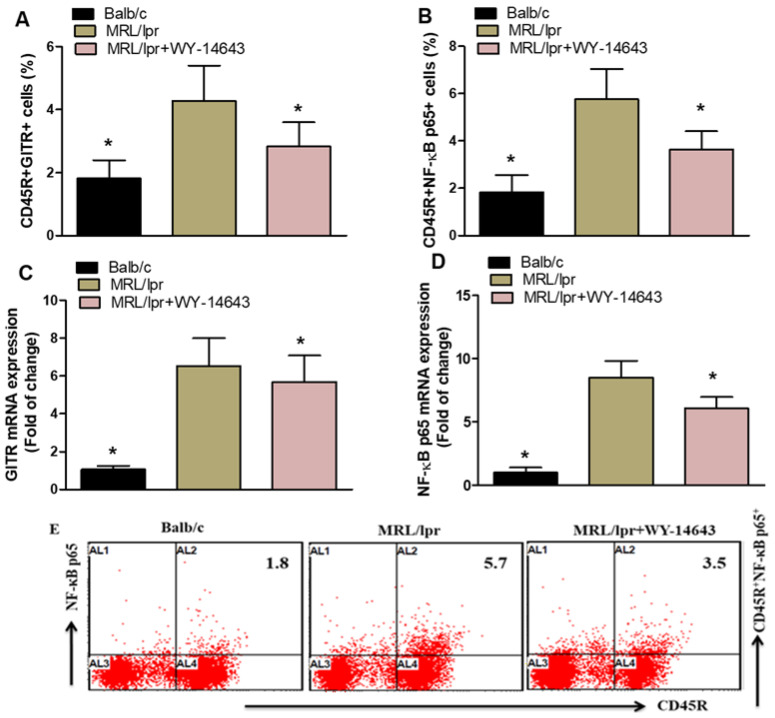
(**A**,**B**). Effects of WY14643 on GITR- and NF-κB p65-expressing CD45R^+^ cells were analyzed by flow cytometry in splenocytes. (**C**,**D**) mRNA levels of GITR and NF-κB p65 were analyzed by RT-PCR in kidney tissue from MRL/lpr mice treated with WY14643. Cells were gated on forward- and side-scatter dot plots to determine the percentage of GITR- and NF-κB p65-expressing CD45R^+^ B cells in the spleen. (**E**) Representative Dot plots of a mouse from each group. MRL/lpr mice were treated with 10 mg/kg WY14643 intraperitoneally daily for eight weeks, whereas Balb/c mice (wild-type control) received a vehicle. The graphs represent the mean score ± SD of six mice per group. Statistical significance was determined as * *p* < 0.05.

Treatment of MRL/lpr mice with WY-14643 significantly reduced the proportions of CD45R^+^GITR^+^ and CD45R^+^NF-κB p65^+^ cells compared with vehicle-treated controls (Figure 5A,B). Likewise, WY-14643 also lowered the mRNA levels of GITR and NF-κB p65 in these mice (Figure 5C,D). Overall, these findings suggest that WY-14643 has anti-inflammatory effects in MRL/lpr mice.

## 3. Discussion

SLE is a long-term, multisystem autoimmune disease characterized by immune dysregulation, autoantibody production, and widespread inflammation [47,48]. Abnormal B cell activation, autoantibody secretion, and immune complex deposition in target organs are key pathological mechanisms of SLE [49]; therefore, targeting B cells is expected to be a treatment for SLE. Our previous research indicated that the PPAR-α agonist WY14643 has anti-inflammatory effects in SLE [50]. CD45R encodes a transmembrane protein-tyrosine phosphatase present on many immune cells [51]. Recent studies suggest that targeting CD45R can modulate immune responses; for example, anti-CD45R antibodies promote regulatory B cells and reduce inflammation [52]. Moreover, anti-CD45R therapy alleviated kidney damage in SLE models, improving BUN, Scr, dsDNA IgG levels, glomerular IgG and C3 deposits, and inflammatory cytokines [53]. Consistent with these findings, our study shows that WY14643 enhances immune regulation in MRL/lpr mice by reducing the levels of several inflammatory mediators.

IFN-γ signaling enhances B cell proliferation during the initial proliferative response after primary antigen exposure [54]. A previous study has shown that the IFN-γ gene signature appears early in SLE [34] and plays a crucial role in lupus nephritis [55]. Some studies indicate that levels of IFN-γ and its related genes are strongly associated with type I IFN activation in patients with SLE [56]. Another study highlights the significant role of IFN-γ in the early and active phases of SLE [13]. Another study has demonstrated that serum IFN-γ levels are higher in patients with SLE than in healthy individuals [57]. WY14643 significantly reduced IFN-γ and IL-6 levels in CD45R^+^ B cells, as measured by flow cytometry, and decreased their mRNA levels in kidney tissue. Since IFN-γ and IL-6 are crucial in lupus development, driving B cell activation, autoantibody production, and inflammation, this reduction suggests that activating PPAR-α may reduce systemic and renal inflammation. These results support earlier studies indicating that PPAR agonists inhibit cytokine production by repressing inflammatory gene transcription.

Both human and animal studies demonstrate that iNOS overexpression is associated with autoimmune diseases [58]. In MRL/lpr mice, an iNOS inhibitor has been shown to prevent glomerulonephritis [59], suggesting that iNOS contributes to the development and progression of SLE. Additionally, recent research in MRL/lpr mice indicates that iNOS promotes the proliferation of T follicular helper cells [60], which are believed to play a key role in SLE pathogenesis by stimulating B cells to produce more IgG [61,62]. Several chemokines participate in the development of lupus nephritis. MCP-1 is linked to kidney damage in SLE [63]. Research indicates that MCP-1-induced protein (IP-10) is significant in the pathogenesis of the disease [64]. Additionally, serum MCP-1 levels are elevated in patients with lupus nephritis [65]. The reductions in iNOS and MCP-1 support WY14643’s anti-inflammatory effects. iNOS promotes oxidative stress and tissue damage in lupus nephritis, while MCP-1 is a key chemokine that recruits monocytes to inflamed areas. The decreased levels of these mediators in splenic B cells and kidney tissue suggest that WY14643 may help prevent leukocyte infiltration and lessen kidney injury. This combined impact on cytokines and chemokines underscores the wide-ranging immunomodulatory potential of PPAR-α activation.

In SLE, abnormal immune activation drives chronic inflammation and organ damage [66]. IL-2 contributes to this harmful process by promoting immune responses that exacerbate autoimmunity. It promotes the growth and activation of autoreactive CD4^+^ T cells [67], which in turn drive B cell differentiation and autoantibody production, key aspects of SLE pathology. Moreover, IL-2 enhances the cytotoxic functions of CD8^+^ T cells [68,69], thereby contributing to tissue damage in organs such as the kidneys and skin. IL-1 has been linked to the development of several autoimmune inflammatory diseases, such as SLE [70]. WY14643 also decreased IL-1α^+^ and IL-2^+^CD45R^+^ cells, along with IL-2 mRNA levels in the kidney. IL-1α is a potent pro-inflammatory cytokine that causes tissue damage, whereas IL-2 has a complex regulatory role in T and B cells. In SLE, dysregulated IL-2 signaling impairs immune tolerance. The decline in these cytokines indicates that WY14643 might help restore immune balance and reduce inflammation in SLE.

Previous research has identified a pathogenic role for Notch signaling in SLE [71,72]. Notably, Zhang et al. showed that inhibiting Notch1 signaling improves murine lupus triggered by activated lymphocyte-derived DNA by preventing macrophage M2b polarization [30]. A recent study also demonstrated that blocking Notch1 signaling significantly reduces the development of pristane-induced murine lupus, underscoring the benefit of targeting Notch1 in SLE [35]. Notch3 regulates epithelial and inflammatory responses, facilitating the occurrence of acute kidney injury [73]. Activation of the Notch-3 receptor promotes inflammation and fibrosis after tubulointerstitial kidney damage [74]. The previously noted proinflammatory role of Notch-3 may therefore be linked to these acute kidney injuries [75]. WY14643 significantly inhibited the Notch1 and Notch3 signaling pathways. Notch signaling promotes B cell activation, differentiation, and survival, processes that are abnormally heightened in lupus. The suppression of Notch 1 and Notch 3 in both splenic B cells and kidney tissue suggests that activating PPAR-α could disrupt these harmful B cell signaling pathways. This is particularly significant because Notch signaling has been linked to autoantibody production and the development of lupus nephritis.

NF-κB activation promotes the production of inflammatory mediators that worsen disease symptoms [76]. The NF-κB signaling pathway is well established as being activated in SLE, and its excessive activation has been linked to disease development and progression [77]. The SLE group showed higher NF-κB expression than the control group [78]. GITR messenger RNA levels are elevated in peripheral blood mononuclear cells from patients with SLE [79,80]. Additionally, a study confirmed that patients with active SLE exhibit higher GITR expression [81,82]. Finally, WY14643 reduced the expression of GITR and NFκB p65, two molecules central to immune activation and the transcription of inflammatory genes. NFκB p65 is a master regulator of inflammatory responses, and its inhibition suggests a key mechanism through which WY14643 suppresses cytokine and chemokine expression. GITR, a co-stimulatory receptor, enhances B cell activation and survival; its downregulation further supports the immunosuppressive effects of PPAR-α agonism.

Overall, these findings demonstrate that WY14643 exerts multiple anti-inflammatory effects in MRL/lpr mice by reducing cytokine production, lowering chemokine levels, and disrupting key B cell activation pathways. The consistent reduction in inflammatory mediators in both spleen and kidney tissues indicates that PPAR-α agonists could be effective for managing systemic inflammation in SLE. By modulating pathways such as NF-κB and Notch signaling, WY14643 may offer a distinct mechanistic approach to controlling autoimmune responses. This study shows that WY14643 decreases inflammatory mediators in splenic CD45R^+^ B cells and reduces expression of cytokines and signaling molecules in the kidneys of MRL/lpr mice.

We acknowledge a few limitations in our research. First, the experiments rely solely on a pharmacological PPAR-α agonist. Although WY14643 is commonly used to activate PPAR-α, such agents can have off-target effects, so we cannot definitively say that the anti-inflammatory effects are solely due to PPAR-α signaling. Second, although we observe decreased levels of inflammatory mediators in CD45R^+^ B cells and kidney tissue, the study does not examine other immune cells involved in SLE development, such as T cells, dendritic cells, and macrophages. Another limitation of our study is that renal mRNA expression was assessed using whole-kidney homogenates. Therefore, modulation of renal B cells or other immune signaling pathways could not be specifically assessed using immunohistochemistry, immunofluorescence, or targeted cell isolation techniques. Therefore, future studies will use immunohistochemistry, immunofluorescence, or cell-specific isolation techniques.

## 4. Materials and Methods

### 4.1. Chemicals and Antibodies

WY14643 was purchased from Tocris Bioscience in Bristol, UK. Reagents such as RPMI-1640 medium, ionomycin, and Phorbol 12-myristate 13-acetate were purchased from Sigma-Aldrich (St. Louis, MO, USA). The FcR blocking reagent fluorescently labeled antibodies, including CD45R, IFN-γ, IL-6, iNOS, MCP-1, IL-1α, IL-2, Notch-1, Notch-3, GITR, and NF-κB p65; as well as buffers for RBC lysis, permeabilization, fixation, and the were purchased from BioLegend (San Diego, CA, USA); BD Biosciences (San Diego, CA, USA); Thermo Fisher Scientific (Branchburg, NJ, USA); and Miltenyi Biotech (Bergisch Gladbach, Germany). cDNA synthesis kit, and SYBR^®^ Green were purchased from Applied Biosystems (Foster City, CA, USA) and TRIzol^®^ reagent from Life Technologies (Carlsbad, CA, USA).

### 4.2. Animal Experiments

Female MRL/lpr mice and Balb/c mice (wild-type [WT]) were purchased from Jackson Laboratories (Bar Harbor, ME, USA); eight-week-old mice weighing 25–30 g were used. All animals were housed in a specific-pathogen-free environment and provided with ample water and food under a 12 h light–dark cycle, with 6 mice per cage. The laboratory environment was maintained at 21–23 °C with a relative humidity of 50–70%. All animal experiments were approved by the Laboratory Animal Ethics Committee of King Saud University (approval no: KSU-SE-23-02) and performed in accordance with the ARRIVE guidelines 2.0 and the King Saud University Animal Committee guidelines and adhered to the principles of laboratory animal care.

### 4.3. Drug Treatment of Animals

Eight-week-old MRL/lpr mice and WT mice were randomized into three groups (*n* = 6 per group). The sample size for the present investigation has been determined based on our prior knowledge and expertise with these experiments. In all experimental studies, the Balb/c group received vehicle, the MRL/lpr group received vehicle, and the MRL/lpr + WY14643 group received 10 mg/kg of WY14643 intraperitoneally daily for eight weeks. The dosage of WY14643 (10 mg/kg, i.p.) was chosen based on previous research [50,83,84,85]. The volume of drugs administered to each mouse was based on its body weight. Subsequently, the mice were euthanized by intraperitoneal injection of pentobarbital sodium (100 mg/kg), followed by cervical dislocation, and samples were collected for subsequent experiments, including flow cytometry staining and RT-PCR analysis.

### 4.4. Flow Cytometric Analysis

For flow cytometry analysis, conjugated antibodies targeting CD45R, IFN-γ, IL-6, iNOS, MCP-1, IL-1α, IL-2, Notch-1, Notch-3, GITR, and NF-κB p65 were used. The following conjugated antibodies (from BioLegend and Santa Cruz Biotechnology, Dallas, TX, USA) labeled spleen cells: FITC anti-mouse CD45R (BioLegend Cat. No. 103206), APC anti-mouse CD45R (BioLegend Cat. No. 103212), PE/Dazzle™ 594 anti-mouse IFN-γ (BioLegend Cat. No. 505845), APC anti-mouse IL-6 (BioLegend Cat. No. 504508), PE anti-mouse iNOS (BioLegend Cat. No. 696806), APC anti-mouse MCP-1 (BioLegend Cat. No. 505910), PE anti-mouse IL-1α (BioLegend Cat. No. 503203), APC anti-mouse IL-2 (BioLegend Cat. No. 503809), APC anti-mouse Notch-1 (BioLegend Cat. No. 130613), Alexa Fluor^®^ 647 anti-mouse Notch-3 (BioLegend Cat. No. 130511), PE/Cyanine7 anti-mouse GITR (BioLegend Cat. No. 126317), and FITC anti-mouse NF-κB p65 (Santa Cruz Biotechnology Cat. No. SC-8008). Splenocytes were treated with PMA/ionomycin (Sigma-Aldrich) and Golgi-plug (BD Biosciences) for 4 h before labeling [86]. After washing, surface CD45R staining was performed, followed by fixation and permeabilization. Fluorescent antibodies against IFN-γ, IL-6, iNOS, MCP-1, IL-1α, IL-2, Notch-1, Notch-3, GITR, and NF-κB p65 labeled the spleen cells. The flow cytometry gating strategy involved first gating singlets based on FSC-A versus FSC-H, then removing dead cells and debris. Live lymphocytes were identified on FSC-SSC plots, and CD4^+^ T cells were gated. Within this subset, intracellular cytokine expression (IFN-γ, IL-17A, and IL-9) was assessed. To determine the different inflammatory mediators in lymphocytes, lymphocytes were separated from other immune cells (monocytes and granulocytes) using a conventional gating strategy based on physical properties (forward and side scatter). Lymphocytes were identified on FSC-SSC plots, and CD45R^+^ cells were gated. The percentages of other immunological markers were then analyzed in CD45R^+^ cells within their respective gates. The percentage of CD45R^+^IFN-γ^+^, CD45R^+^IL-6^+^, CD45R^+^iNOS^+^, CD45R^+^MCP-1^+^, CD45R^+^IL-1α^+^, CD45R^+^IL-2^+^, CD45R^+^Notch1^+^, CD45R^+^Notch3^+^, CD45R^+^GITR^+^, and CD45R^+^NF-κB p65^+^ cells were determined in the lymphocyte gate. A Beckman Coulter FC500 flow cytometer (Indianapolis, IN, USA) collected 10,000 cell events, and data were analyzed using CXP software version 2.0 [86].

### 4.5. RT-PCR Analysis

Total RNA was isolated from kidney tissue using TRIzol reagent (Life Technologies, Paisley, UK). cDNA synthesis was performed with a high-capacity cDNA reverse transcription kit, followed by real-time PCR with SYBR^®^ Green PCR master mix (Applied Biosystems (Foster City, CA, USA), according to the manufacturer’s instructions. Primers were selected from PubMed. The assay primers included: IFN-γ (forward: 5′-CGGCACAGTCATTGAAAGCC-3′, reverse: 5′-TGCATCCTTTTTCGCCTTGC-3′), IL-6 (forward: 5′-GCCTTCTTGGGACTGATGCT-3′, reverse: 5′-GACAGGTCTGTTGGGAGTGG-3′), iNOS (forward: 5′-TCAGCCAAGCACTCCAATGT-3′, reverse: 5′-AGTGATGGAGGTGCCCTAGT-3′), MCP-1 (forward: 5′-CAAAGCCAGGGGCCTTTTTC-3′, reverse: 5′-TACCAGGAGCCAGGCATAGT-3′), IL-2 (forward: 5′-GGAACCTGAAACTCCCCAGG-3′, reverse: 5′-AATCCAGAACATGCCGCAGA-3′), Notch-1 (forward: 5′-GCCTCAAGCCCCTGAAGAAT-3′, reverse: 5′-GCGCTTTCGACGATCTGAAC-3′), Notch-3 (forward: 5′-AGGCCATGGTCTTCCCCTAT-3′, reverse: 5′-ACCTCCCCCATCAGACTCTC-3′), GITR (forward: 5ʹ-CCAAGCCAGACGCTACAAGA-3′, reverse: 5ʹ-CAGGGAAGGGTGCAGAACAT-3′), NF-κB p65 (forward: 5′-CTTCTCTATGGCGTCGTCCC-3′, reverse: 5′-AAGATGGCCTCCTTCACAGC-3′), and GAPDH (forward: 5′-GGCAAATTCAACGGCACAGT-3′, reverse: 5′-TGAAGTCGCAGGAGACAACC-3′). mRNA levels were normalized to GAPDH as an endogenous reference gene [87], and results are expressed as fold change.

### 4.6. Statistical Analysis

Data are presented as mean ± SD. The data undergo normality and homogeneity tests before analysis. Differences among the three groups were analyzed using one-way ANOVA, followed by Tukey’s post hoc test for multiple comparisons. All statistical analyses were conducted using GraphPad Prism 8; *p* < 0.05 indicated statistical significance.

## Data Availability

The original contributions presented in this study are included in the article. Further inquiries can be directed to the corresponding author.
